# Primates in Human-Modified and Fragmented Landscapes: The Conservation Relevance of Modelling Habitat and Disturbance Factors in Density Estimation

**DOI:** 10.1371/journal.pone.0148289

**Published:** 2016-02-04

**Authors:** Nathalie Cavada, Claudia Barelli, Marco Ciolli, Francesco Rovero

**Affiliations:** 1 DICAM Department of Civil, Environmental and Mechanical Engineering, University of Trento, Trento, Italy; 2 Tropical Biodiversity Section, MUSE—Museo delle Scienze, Trento, Italy; 3 Biodiversity and Molecular Ecology Department, Research and Innovation Centre–Fondazione Edmund Mach, San Michele all'Adige (TN), Italy; 4 Udzungwa Ecological Monitoring Centre, Udzungwa Mountains National Park, Mang’ula, Tanzania; University of South Carolina, UNITED STATES

## Abstract

Accurate density estimations of threatened animal populations is essential for management and conservation. This is particularly critical for species living in patchy and altered landscapes, as is the case for most tropical forest primates. In this study, we used a hierarchical modelling approach that incorporates the effect of environmental covariates on both the detection (i.e. observation) and the state (i.e. abundance) processes of distance sampling. We applied this method to already published data on three arboreal primates of the Udzungwa Mountains of Tanzania, including the endangered and endemic Udzungwa red colobus (*Procolobus gordonorum*). The area is a primate hotspot at continental level. Compared to previous, ‘canonical’ density estimates, we found that the inclusion of covariates in the modelling makes the inference process more informative, as it takes in full account the contrasting habitat and protection levels among forest blocks. The correction of density estimates for imperfect detection was especially critical where animal detectability was low. Relative to our approach, density was underestimated by the canonical distance sampling, particularly in the less protected forest. Group size had an effect on detectability, determining how the observation process varies depending on the socio-ecology of the target species. Lastly, as the inference on density is spatially-explicit to the scale of the covariates used in the modelling, we could confirm that primate densities are highest in low-to-mid elevations, where human disturbance tend to be greater, indicating a considerable resilience by target monkeys in disturbed habitats. However, the marked trend of lower densities in unprotected forests urgently calls for effective forest protection.

## Introduction

Knowledge on abundance and distribution of animal species is required when planning for conservation actions [1–3]. In this context, primates are excellent study subjects as they represent good ecological indicators in tropical rainforest, being highly sensitive to habitat changes, hunting and other forms of disturbance [4–6]. Indeed they are the mammal order with the highest proportion of species under threat [7,8], due to the effect of different drivers [9,10], that often interplay following complex and site-specific patterns [11]. Ideally therefore, proper estimation of population densities should accurately account for potential covariates, including spatially-explicit ones, that can help to understand how ecological processes are involved in the high spatial heterogeneity of population abundance, as well as to understand how these populations will respond to environmental changes [3,12]. In this perspective, modelling the spatial patterns of threatened populations at a landscape-level can be very informative, particularly when considering species that occupy highly diverse habitats [13–15]. Such approach is also of clear conservation relevance for site prioritization, i.e. to identify the main drivers of change in variation of species density and locate those areas that need urgent intervention [16].

Meanwhile, it is widely acknowledged that models of animal density and their habitat preferences need to consider imperfect detectability of species at occupied sites [17–19], to avoid incorrect estimates and predictions [20,21]. This is particularly relevant for primates for which population assessments are inherently complex because of the habitat characteristics [17,22], and their social structure [23]. Hence, the use of the ‘canonical’ application of distance sampling [24], i.e. one that does not consider the differential influence of covariates on abundance and detection, may not be the most informative approach when analyzing density of primates that occupy heterogeneous landscapes. Here, we address this issue by providing an application of the hierarchical modelling framework by Royle, Dawson and Bates [25], that allows to include covariates both in the observation (detection) and in the state (abundance) processes.

We applied such method to distance sampling data collected in the Udzungwa Mountains of Tanzania, an outstanding hotspot for primate diversity and endemism in Africa, where relevant background work has been already conducted on primates. We targeted three species of arboreal monkeys, including the endemic and threatened Udzungwa red colobus (*Procolobus gordonorum*). Previous studies by Araldi *et al*. [26] applied the conventional distance sampling approach and, even though these authors realized a robust survey effort for well-informed density estimates, they did not consider the relationship between densities and environmental covariates. Barelli *et al*. [27] presented an assessment of primates’ responses to habitat factors and human disturbance using the observed encounter rate of primate social groups as the response variable in a multivariate regression framework. Hence, they did not account for imperfect detection. Both studies provided informative results regarding contrasting density estimates among forest blocks [26] and the consistent influence of elevation and climber coverage on the encounter rate of primates [27]. However, further investigation using a spatially-explicit, inferential framework is highly relevant to understand how habitat and disturbance covariates affect density and detectability. The objectives of our study were: 1) to obtain species-specific models from distance sampling data, using an approach that has rarely but successfully been applied to derive the abundance of endangered animal populations [20,28,29]; 2) to assess if such selected models could improve the sensitivity of estimates of primates population density; 3) to gain relevant information for conservation purposes by modelling the spatial variation of primate density in a highly heterogeneous and complex human-natural system.

## Materials and Methods

### Ethics statement

Data collection did not involve direct contact or interaction with the animals. We analyzed data collected by earlier studies [26,27] in respect and under permissions of the relevant authorities indicated therein.

### Study area and species

**Fig 1:**
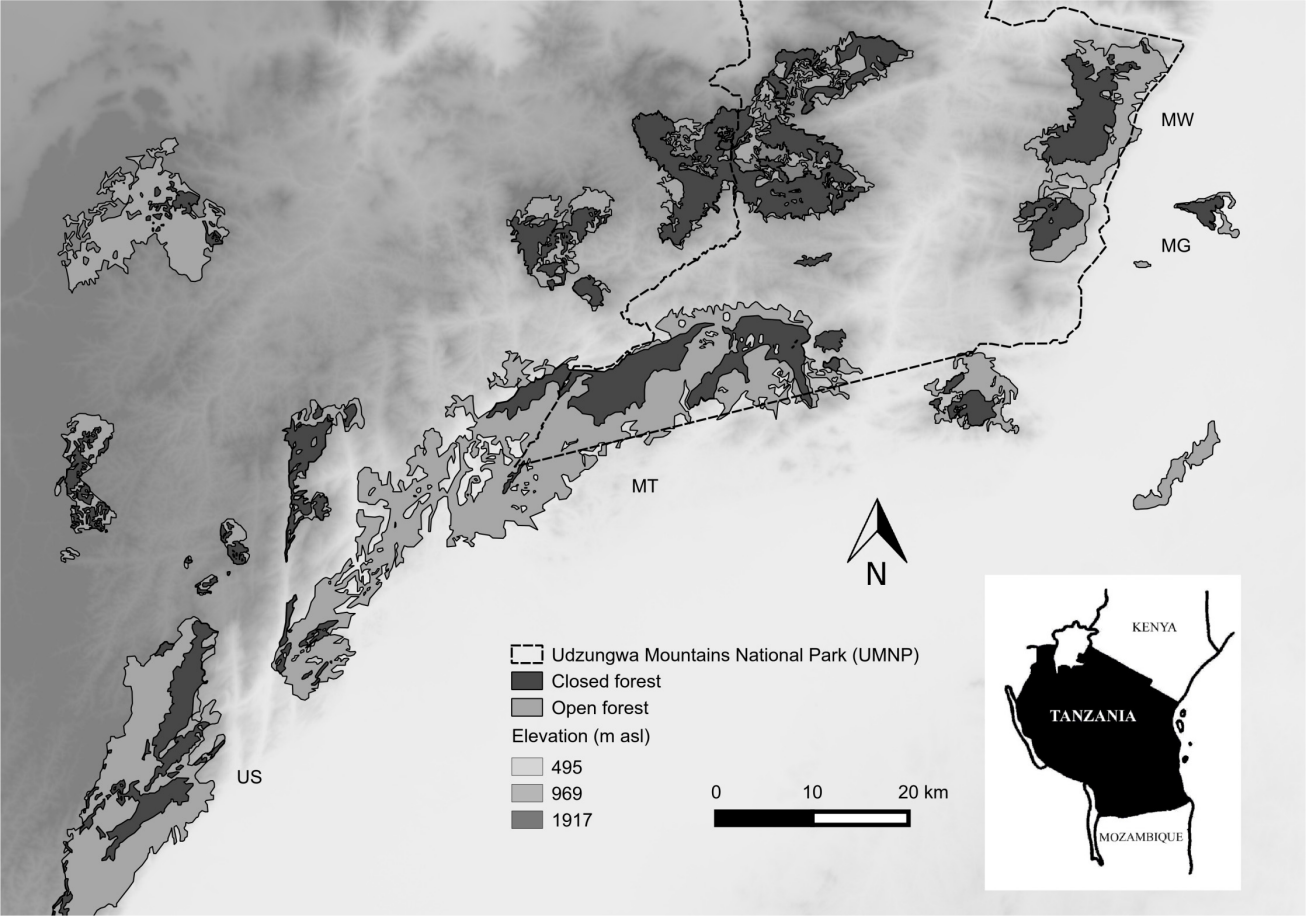
Udzungwa Mountains National Park Map. Map of the Udzungwa Mountains National Park, Tanzania, showing the four forests surveyed (Magombera, MG; Matundu, MT; Mwanihana, MW and Uzungwa Scarp, US) for primate density data collection.

The Udzungwa Mountains (7°40'–8°40' S, 35°10'–36°50' N; Fig 1) extend over >19,000 km² [30] and represent the southern block of the Eastern Arc Mountains of Kenya and Tanzania [26,31], within the Afromontane biodiversity hotspots [32]. The mountains are characterized by the presence of several forest blocks that differ in elevation range (290–2,500 m a.s.l.), area (from 12 to >500 km²), habitat type and protection level [26,33].

Data were collected by Araldi *et al*. [26] and Barelli *et al*. [27] in four different forest blocks, namely Magombera (MG), Matundu (MT), Mwanihana (MW) and Uzungwa Scarp (US), with MG and US showing intense human disturbance due to the absence of legal protection [34,35].

The study focused on three species of arboreal primates that show a widespread distribution across the Udzungwa Mountains: the Peters' angolan colobus (*Colobus angolensis palliatus*) (henceforth BW), the endemic and endangered (IUCN, 2011) Udzungwa red colobus (hencefort RC) and the Tanzania Sykes' monkey (*Cercopithecus mitis monoides*) (henceforth SY).

We refer to Barelli *et al*. [27] and Araldi *et al*. [26] for detailed information about the study area and species.

### Data set: primates and habitat covariates

**Fig 2:**
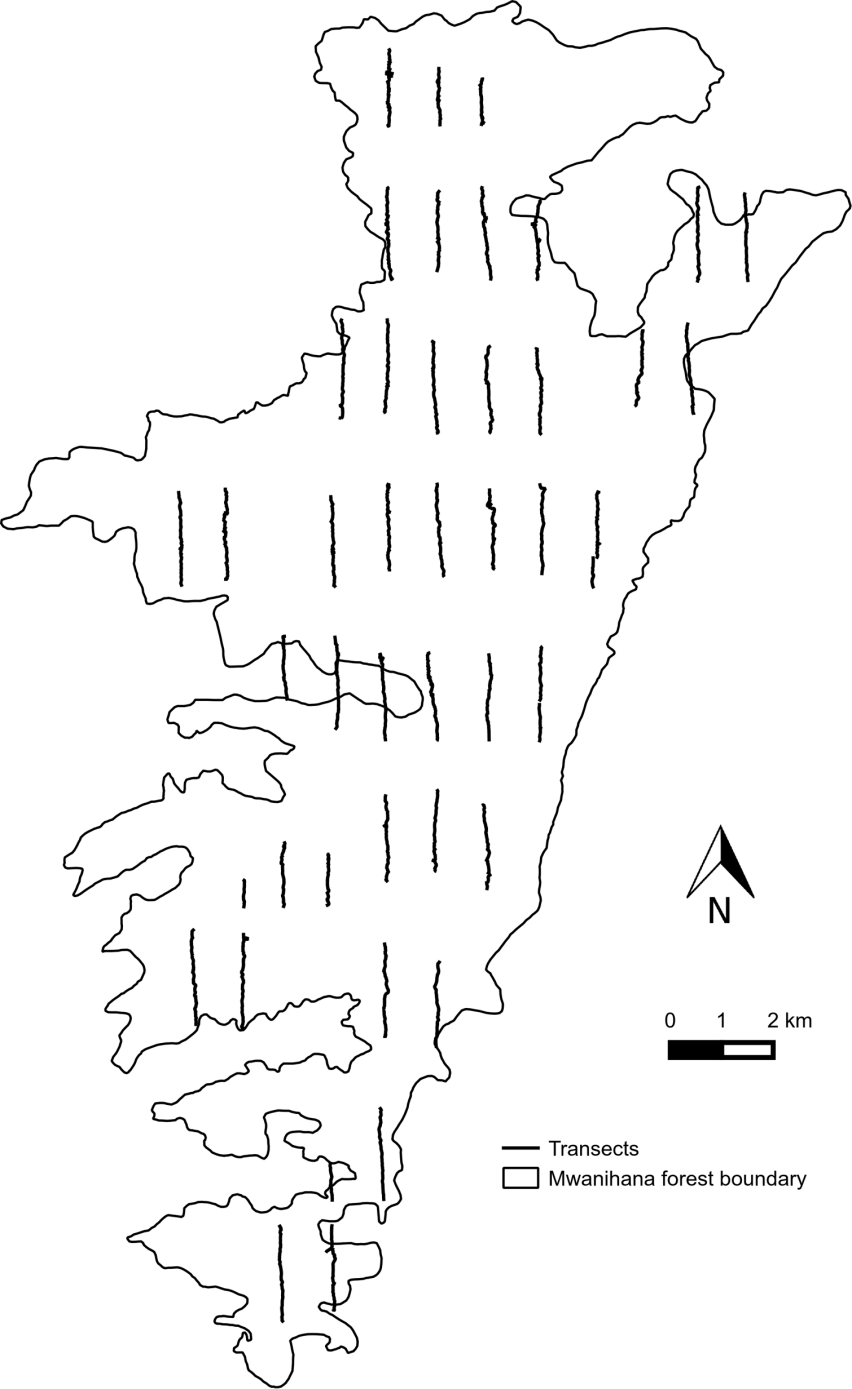
Sampling grid in Mwanihana forest. Map of Mwanihana forest (MW) with the sampling grid, as an example of diffused grid of transects walked for primate density estimations in Udzungwa Mountains National Park of Tanzania.

We used data in Araldi *et al*. [26] and Barelli *et al*., [27] that were collected through systematic line transects following the standardized distance sampling approach [24]. Authors achieved a uniform coverage of target forests (Fig 2). Arboreal vegetation and disturbance parameters were collected by establishing four squared vegetation plots, 25 by 25 m each, centered on each line transect, with a total of 176 plots sampled (see [27]).

### Statistical method

We modeled the observed data as a hierarchical coupled logistic regression. One step of the modelling process is related to the partially observed true state (occurrence, the result of a biological process); the other step describes detection, that is the result of both the biological process and the observation process (i.e. how animals are detected). In detail we assumed animals’ abundance at transect level to have a Poisson distribution (*Xi* ~ Poisson (*λi*); *i* = 1,…,*n*), with *λ* being the expected value of *X* (*λ = E(x)*). We modelled the detection process according to a multinomial distribution and we expected the detection probability to monotonically decrease with the increasing distance from the observer, as per conventional distance sampling theory [24]. We verified this process by looking at the histograms of the distance records. We removed outliers from the data-set, defining a species-specific right- truncation distance, looking at the right tail of the plotted distance frequency distribution. We set such truncation distance at 100m for BW and SY and at 90m for RC. Observations taken at larger distances were scarce and provided little information for the estimation of the detection function [24]. In detail we removed 64 outliers for RC and SY and 67 outliers for BW. We noted heaps mainly in the first distance class, suggesting that rounding errors were mainly close to distance = 0. We therefore grouped in intervals distances that were recorded on a continuous scale, correcting for heaping and to improve estimates of density and better model fit [24]. Thus, we defined 5 bins of 20 m for the analysis on BW, 6 bins of 15 m for the analysis on RC and 4 bins of 25 m for the analysis on SY.

Using the function ‘*distsamp*’ in R package ‘*unmarked’* [36] we modelled data separately for each of the three primate species. We first checked the performance of different detection functions (uniform, half-normal and hazard-rate) on the simplest model, without considering the covariates effect. Based on the Akaike Information Criterion (AIC), we retained the half-normal function *g(y)* = exp—(*y* 2/2 *σ* 2), with *y* being the distance class and with *σ* being the scale parameter for the detection function. We then incorporated in the model the influence of transect-specific covariates on both *λ* and *σ*, using a log link function. We built models using all the possible combinations of environmental and human-disturbance variables, sampled at the transect level, to determine how they affect both the detection process and the presence of the animals, based on an set of assumptions (Table 1); see also [27]. In addition to distance which is an inherent covariate of the detection process, we assumed detection to be influenced by the following covariates: (1) group size, assuming that larger groups are more easily detected in the canopy at larger distances [24]; (2) forest block, as a nominal covariate representative of the heterogeneity among forests, given that each forest is a discrete area sampled; (3) canopy cover and (4) percentage of climbers; (5) steepness and (6) distance to anthropic disturbance (i.e. roads and villages). We used these covariates also when modelling the state process, in addition to (7) altitude, (8) diversity of tree communities, calculated as the Simpson's reciprocal diversity index; we also considered, as proxies of disturbance, (9) count of signals of human presence along transects (cutting signs, recent and old paths, and trails made by humans, sites where pit sawing had been carried out or charcoal was produced, as well as signs of recent and old poacher camps, incidence of animal snares) and (10) distance from the forest edge.

**Table 1:** List of the covariates sampled in the four forest blocks of the Udzungwa Mountains, Tanzania.

Habitat variables	Variable effect	Hypothesized relationship with the detection process
*Covariates on detection*		
Forest block	no interpretation	Highly diverse morphology in each forest block, natural or human driven.
Group size	+	Large groups are more easily detected even at larger distances [23].
Canopy cover	-	Closed canopy area reduce visibility.
Distance from disturbance	-	Proximity to human disturbance and therefore to disturbed habitats can facilitate animal detection.
Percentage of climbers	+	Climbers are representative of areas that have been logged in the past and are found in lowland regenerating forests [46,47]; being proxies of open habitats they can allow better detection.
Steepness	+	A steep terrain originates naturally-broken canopy [27] that increases detectability.
*Covariates on density*		
Forest block	no interpretation	High variability among the forests block in terrain morphology, vegetation structure and formal protection level.
Canopy cover	-	Preference by three target species is shown for disturbed habitats with a patchy canopy cover [27].
Total basal area	-	Mature, old-growth forests that present large total basal area values are less preferred [27,37].
Mean basal area	+	Colobines are found to selectively feed on large tree species [5], showing high scores for mean basal area.
Simpson diversity index	+	A higher species diversity can represent a greater variety of food sources, thus allowing primates presence [38–40].
Percentage of climbers	+	Vegetation diversity in the tropics is also related to vines and climber species, on which Udzungwa primates rely for a large portion of their dietary requirements [41].
Altitude	-	Lower to mid-elevations are characterized by the presence of semi- deciduous forests where Colobines can find young and more digestible leaves [27]. The frugivorous Sikes' monkeys [42,43], are not found at higher elevations, where fruit productivity is low.
Steepness	+	Steep terrains facilitate moderate climbers spread and colonization (i.e more digestible food items; [44]), due to natural occurring brakes in the canopy.
Human impact	-	Noisy and disturbing human activities such as logging, together with
Distance from edge	+	hunting may affect animals behaviour and can cause avoidance and
Distance from disturbance	+	fleeing responses [40,45].

Covariates were examined in the model building step for the three primate species (BW, RC and SY) and their predicted effect on both the detection and the density processes is reported as (+) (= positive) and (-) (= negative).

We used AIC to rank all the candidate models and we considered as equivalent those models showing ΔAIC<2 [37]. This criterion prevent us from unequivocally define a single best model on which to base predictions. We thus determined Akaike weights (*wi*) for each of the best models (R package *MuMIN*; [38]) and to further reduce ambiguity, we derived the relative importance of each variable, on a scale from 0 to 100. We decided to favor the model with the lowest number of parameters, selecting only the variables that showed an importance of at least 50%. To verify the goodness of fit of the selected model we performed a parametric bootstrapping, simulating 200 datasets from the fitted model and defining a function that returned the fit-statistic of the Pearson's X2. We used non parametric bootstrap to estimate the uncertainty (i.e. SE) of the parameters in the model. We then used the resulting best species-specific models selected, to predict primates group density, as well as their detectability, in each sampled forest block and in each of the plot that were sampled along the transects, for which measurements of the influential habitat variables were available.

We also assessed how the hierarchical structure of our analysis could improve our estimates, by comparing our results with those from Araldi *et al*. [26], and we assumed these authors’ estimates to be comparable with those from our null model, i.e. one that assumes no covariates effect. To test for differences between the two approaches, we used a t-test after assessing normality with Shapiro-Wilk tests [39]).

## Results

After right truncating the data at 100 m we retained 90 observations for BW and 129 for SY, while we retained 97 observations for RC with a 90 m truncation. Detection functions indicated that all assumptions for the method were met, i.e. they showed a monotonic decrease with increasing distance as well as good fit on the observed data. No spikes were present after binning the data in distance classes.

**Fig 3:**
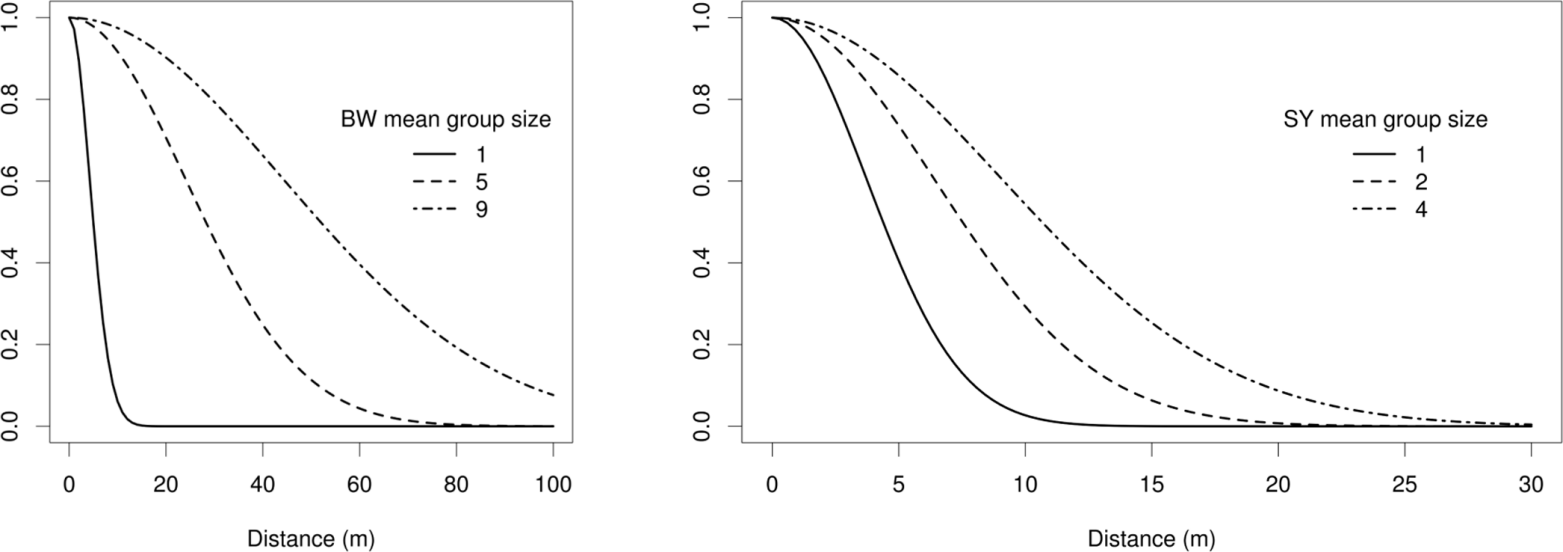
Best selected models detection functions. Detection functions from the best AIC models, shown for the 0.25, 0.50 and 0.75 quartiles of the covariate group size for Peters' Angola colobus (BW) and Tanzania Sykes' monkey (SY).

**Fig 4:**
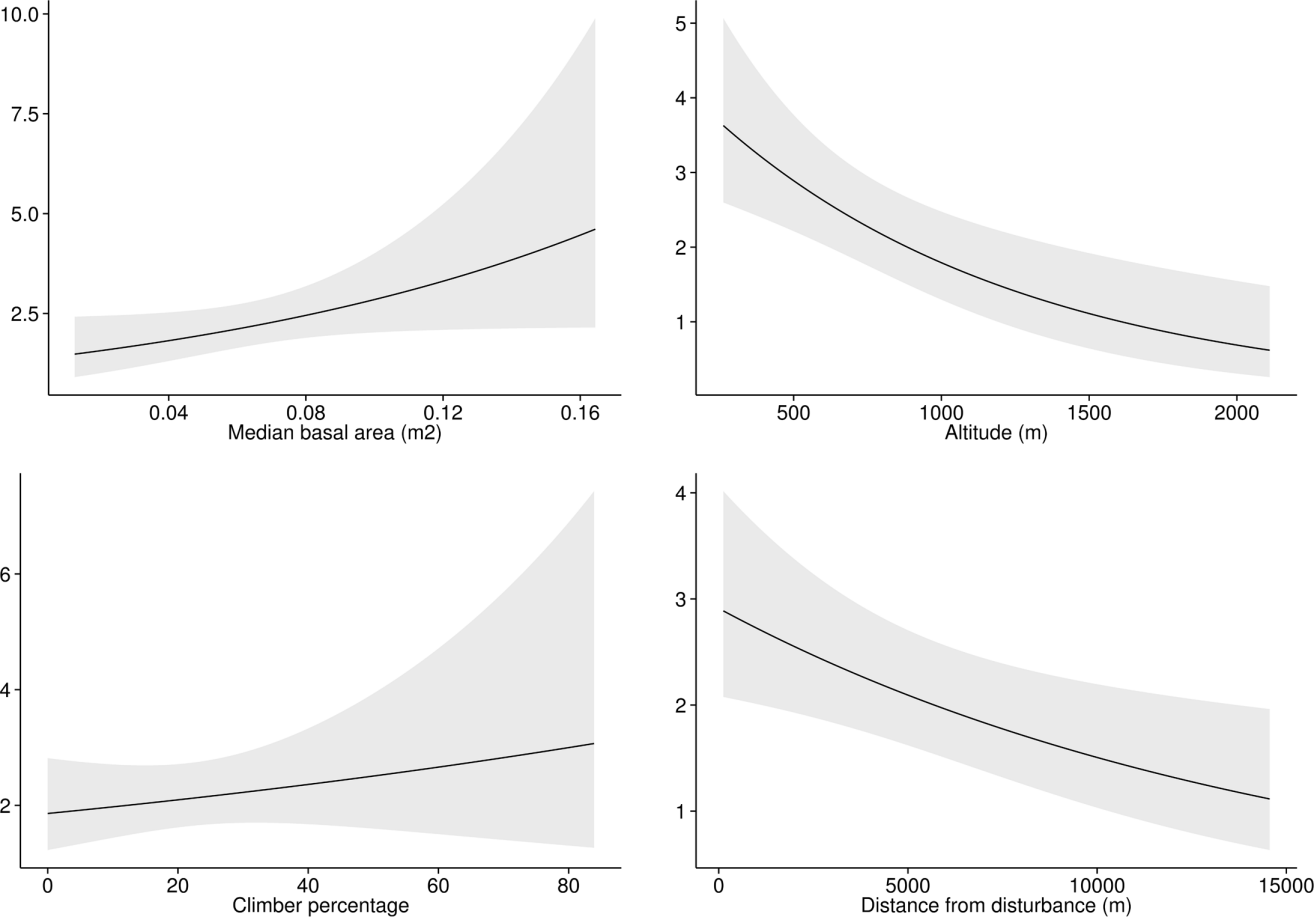
Covariates effect on density estimation. Covariates effect on group density estimation, shown for the best model selected for the Udzungwa red colobus (RC).

Model selection for BW resulted in a model containing an effect of group size (+, i.e. a positive effect) on detection and an effect of percentage of climbers (+), human impact (-, i.e. a negative effect) and forest block on density; the best model for RC contained an effect of forest block, climbers percentage (+) and distance from disturbance (-) on detection and an effect of mean basal area (+), percentage of climbers (+), altitude (-) and distance from human disturbance (-) on density. The best model for SY retained an effect of group size (+) on detection and of climber percentage (+) and altitude (-) on abundance (Tables 2 and 3; Figs 3 and 4; Figures A and B in S1 File).

**Table 2:** Akaike information criterion (AIC) value for high ranked models of primates' density (λ) and the shape parameter (σ) of a half-normal detection function.

Model	AIC	ΔAIC
**Peters' Angola colobus (*Colobus angolensis*)**		
σ(group size)λ(climber% + human impact + forest)	425.84	
σ(group size)λ(climber% + forest)	426.49	0.65
σ(group size)λ(canopy + climber% + simpson^a^ + forest)	428.47	2.63
σ(۰)λ(۰)	533.05	106.561
**Udzungwa red colobus (*Procolobus gordonorum*)**		
σ(forest + dist_disturbance^b^ + climber%)λ(mba^c^ + climber% + altitude + dist_disturbance)	557.41	
σ(forest + dist_disturbance)λ(mba +climber% + altitude + dist_disturbance)	558.25	0.84
σ(forest + dist_disturbance + climber%)λ(mba + climber% + steepness + altitude + dist_disturbance)	558.59	1.18
σ(۰)λ(۰)	603.34	45.93
**Tanzania Sykes' monkey (*Cercopithecus mitis monoides)***		
σ(group size)λ(climber% + altitude)	513.14	
σ(group size + human impact + canopy + climber%)λ(climber% + altitude)	514.45	1.32
σ(group size)λ(climber% + steepness + altitude)	514.55	1.41
σ(۰)λ(۰)	595.96	82.83

a Simpson's reciprocal diversity index

b Distance from anthropic disturbance (i.e. roads and villages)

c Mean basal area

**Table 3:** Parameter estimates and their standard error for the final models selected for the three primate target species that presented the lowest AIC values.

Model and coefficient		CI (95%)	SE
**Peters' Angola colobus**			
Detection (σ)			
Intercept	10.2	10.12–10.2	2.15
Group size	12	11.98–12.06	3.278
Density (λ)			
Intercept	1.42	1.01–1.83	0.692
Climber %	0.2	0.02–0.37	0.192
Human impact	-0.14	-0.36 –-0.08	0.228
Forest Matundu	-0.3	-0.87–0.27	0.473
Forest Mwanihana	-0.35	-0.91–0.2	0.369
Forest Uzungwa Scarp	-0.97	-18.3 –-0.1	0.951
**Udzungwa red colobus**			
Detection (σ)			
Intercept	2.54	1.22–3.87	6.95
Forest Matundu	8.43	-52.13–68.99	7.98
Forest Mwanihana	6.14	-24.36–36.65	7.11
Forest Uzungwa Scarp	-0.87	-1.86–0.12	8.78
Distance from disturbance	-1.78	-3.51 –-0.04	5
Climber %	0.51	-0.17–1.18	4.51
Density (λ)			
Intercept	0.74	0.49–1	1.55
Mean basal area	0.21	0–0.43	0.41
Climber %	0.09	-0.11–0.3	0.63
Altitude	-0.53	-0.83 –-0.22	0.37
Distance from disturbance	-0.27	-0.47 –-0.07	0.44
**Tanzania Sykes' monkey**			
Detection (σ)			
Intercept	6.57	6.53–6.61	1.385
Group size	7.06	7.03–7.08	2.809
Density (λ)			
Intercept	1.28	1.1–1.47	0.117
Climber %	0.16	-0.03–0.35	0.078
Altitude	-0.22	-0.45–0	0.107

**Fig 5:**
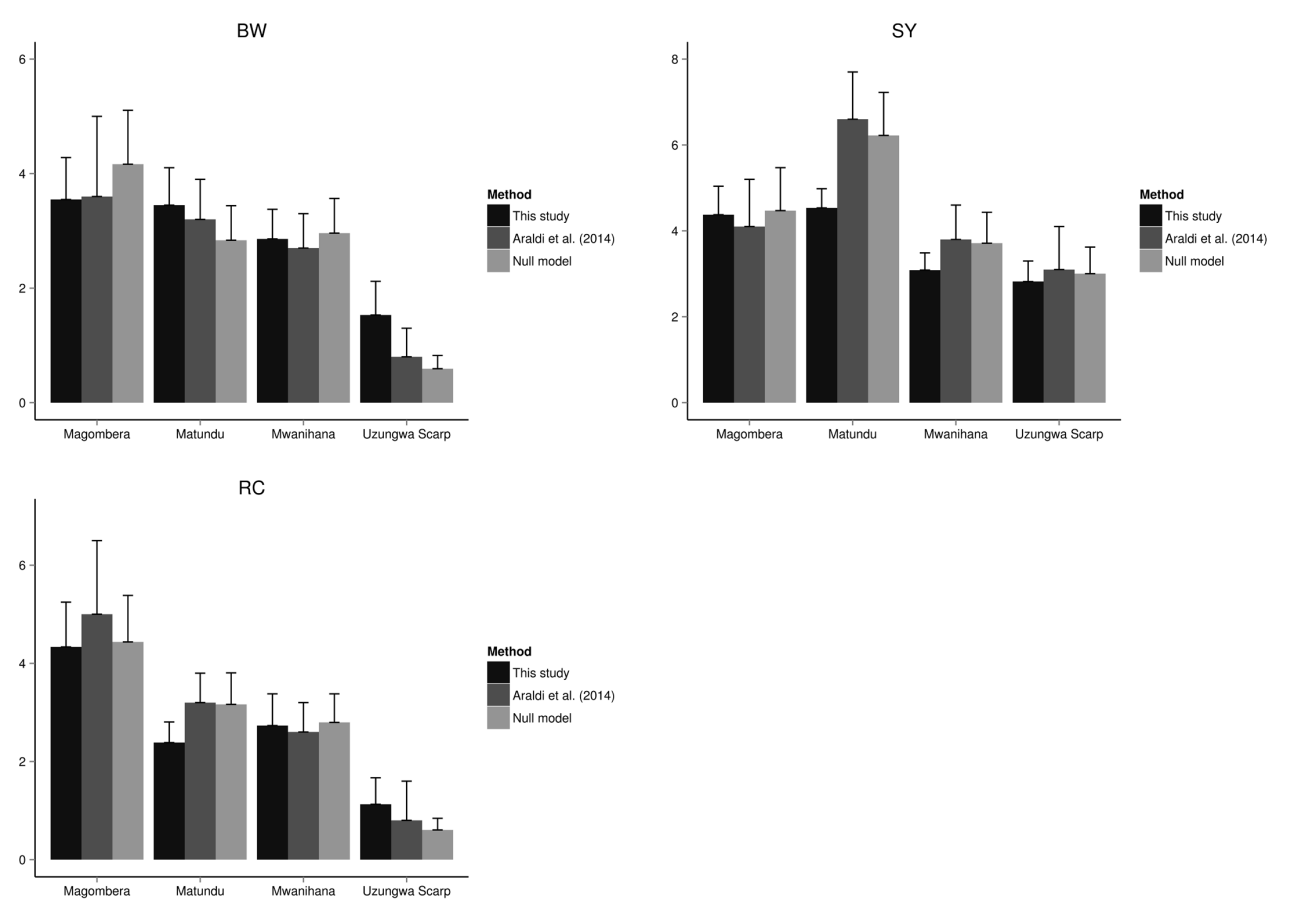
Density estimates comparison between methods. Comparison between the estimated density values for the three primate species (Peters' Angola colobus (BW), Udzungwa red colobus (RC), Tanzania Sykes' monkey (SY)), obtained applying different methods (i.e. hierarchical modelling with covariates (this study); the study by Araldi *et al*. [26]; null model without covariates).

The bootstrap P value based on the Chi-square statistic showed adequate fit for all the species specific models (*P* = 0.94 for BW; *P* = 0.18 for RC; *P* = 0.37 for SY). Testing for differences between density estimates from our null model and estimates in Araldi *et al*. [26] confirmed the equivalence of the two methods (*P* = 0.16). This in turn supports our hypothesis of a better performance (based on delta AIC of models with covariates *vs* null models) of our best models to estimate primates density (Fig 5; Table 4) relative to the conventional approach (ΔAIC = 106.507 for BW; ΔAIC = 45.93 for RC; ΔAIC = 82.83 for SY; Table 2).

**Table 4:** Forest specific values of detectability and groups density for the three primates target species.

Species and forest	Detectability (SE)	Density (groups/km2) (SE)
**Peters' Angola colobus (*Colobus angolensis)***		
Magombera	0.15 (0.01)	3.49 (0.73)
Matundu	0.11 (0.007)	3.45 (0.66)
Mwanihana	0.13 (0.006)	2.9 (0.53)
Uzungwa Scarp	0.04 (0.007)	1.43 (0.57)
**Udzungwa red colobus (*Procolobus gordonorum*)**		
Magombera	0.12 (0.006)	4.88 (0.97)
Matundu	0.17 (0)	2.4 (0.41)
Mwanihana	0.17 (0)	1.83 (0.33)
Uzungwa Scarp	0.06 (0.005)	1.2 (0.34)
**Tanzania Sykes' monkey (*Cercopithecus mitis monoides)***		
Magombera	0.13 (0.01)	4.38 (0.66)
Matundu	0.16 (0.009)	4.53(0.45)
Mwanihana	0.12 (0.01)	3.09 (0.4)
Uzungwa Scarp	0.16 (0.01)	2.82 (0.48)

**Fig 6:**
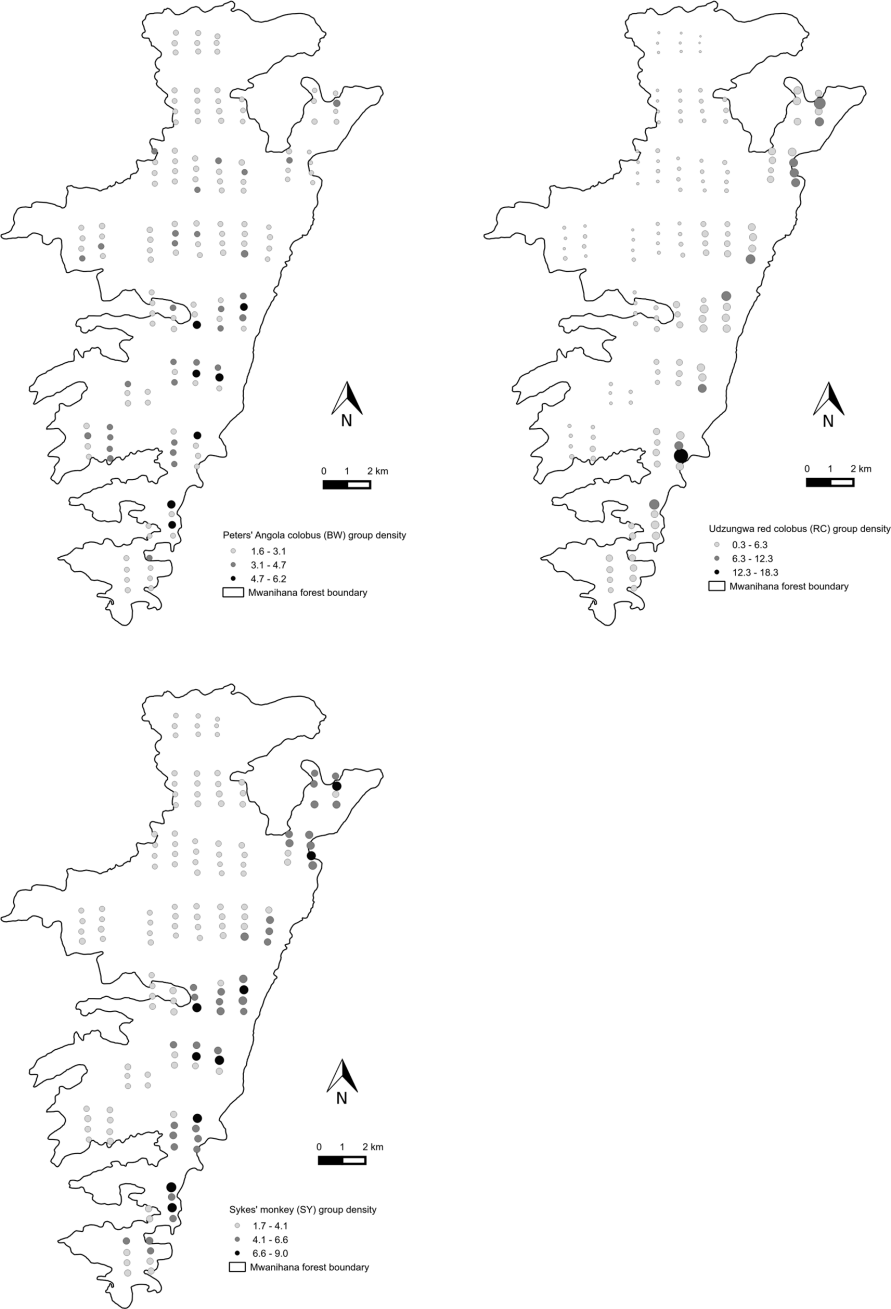
Spatially explicit modelling of animal density. Predicted density (groups/km2) for the three primate species (Peters' Angola colobus (BW), Udzungwa red colobus (RC), Tanzania Sykes' monkey (SY)) from the best selected models (see Table 2) in the forest of Mwanihana. Predicted values were obtained for the plots that were sampled along the transects, for which exact values of the influential covariates were available.

Spatially-explicit maps of estimated density at the plot level are shown in Fig 6 and Figure C in S1 File.

## Discussion

Our study aimed to show the importance of accounting for habitat covariates of primate detectability and abundance in distance sampling studies in complex landscapes. The hierarchical analytical approach allowed us to obtain reliable, informative and spatially-explicit estimates relative to previous studies that did not consider the covariate effect [26] nor abundance estimation with imperfect detection [27]. Moreover, the method we used allows for inference on density outside the sampled area. This is of particular relevance when the variables retained in the modelling are spatially diffused, as it usually applies to those derived from remote sensing.

A first important result is how the species-specific group size influences detection. By using this approach, group size effect could be explicitly evaluated and therefore modelled. On the contrary, in conventional distance sampling group size is regressed on estimated probability of detection. The positive relationship between group size and detection in BW and SY, but not RC, is likely explained by different grouping patterns. The average group size of BW and SY was indeed similar (3.84 and 3.41 respectively) and almost five times lower than of RC (17.03). Groups of RC could have been consequently more easily detected even far from the transect line. Indeed focal studies have shown that RC can average 40 individuals in undisturbed forests such as Mwanihana, while BW and SY average group size is <10 and much smaller for SY [40]. Thus group size represents a critical parameter that needs to be carefully considered to avoid underestimation of animal densities, with particular relevance for species whose social units are small (i.e. <5–10 individuals) as is the case of SY, for which, indeed, the parameter 'group size' had a higher effect on detection. As predicted, we found detectability for RC to be negatively influenced by distance from disturbance. This variable represents a proxy for forest structures that can hamper visibility, such as tall and dense canopy in interior forest. Climber percentage, on the contrary, had a positive association with RC detectability. Even if producing a small effect on the detection process (for climber coverage <75%), moderate presence of climbers constitutes a structure of the sub-canopy layer that is seemingly preferred by arboreal primates (see below).

As for the effect of covariates on animal density, we found the percentage of climbers to have a positive effect for all the three species we examined. This result is in line with findings from Barelli *et al*. [27] and Rovero and Struhsaker [41]; climbers represent a food source [42,43], influence canopy connectivity and provide supports for movements in the canopy [44,45].

We found a negative association between altitude and density of RC and SY. This also matches the findings from previous studies [27,41,46] that explained this result in terms of different food availability along the elevation gradients of the study area. Human impact was found to have a negative association only with BW. Hunting pressure is indeed reported to be targeted mainly on this species, which skin is highly demanded [11]. RC and SY appear less affected by hunting and this differential degree of human impact is reported in several other studies [11,47–49]. Density of RC was related to the mean basal area of trees, that had a positive effect, and to distance from disturbance, with a negative effect, contrary to what we hypothesized. This is in line with results by Rovero and Struhsaker [41] and confirms the preferences shown by the species for larger trees that can be found also at forest edges. Here, even if logging is more intense, productivity of the remaining large trees can still be high [50], thanks to an increase in illumination [51].

We found lower values for group density estimates in the US forest block for all the three species and mainly for BW and RC, for which density values were about the 40% lower in US. Nevertheless, variation in density between US and the other forest blocks was particularly substantial for BW (Table 4), for which the parameter level US was found to have a high negative effect on density estimation. Importantly, the variation in density estimates among forests was almost two times lower than that reported in Araldi *et al*. [26]. Such underestimation may have been smoothed by our analysis because of adding the effect of covariates on both the detection probability and the state process [52,53]. This is of particular conservation relevance in highly disturbed habitats, like US, where animals are sparse and shy, and therefore tend to hide and go undetected relatively more than in other forests (Table 4). In general, our results further confirm that the absence of protection in US highly impacts the colobine monkeys, with pressures that mainly derive from targeted hunting and to lesser extent to habitat degradation [11,54]. These findings in turn support the hypothesis that colobines are more sensitive than Tanzania Sykes' monkeys to highly disturbed habitats and to human impact that deeply affects the structural characteristics of the forest [54–56].

### Conclusions and conservation recommendations

Obtaining reliable and informative estimates of primate density in complex and human-modified landscapes is difficult, yet with habitat degradation and loss being a pan-tropical phenomenon, an increasing proportion of primate species is found in degraded and patchy habitats [15]. Our study demonstrates how the inference on abundance is improved by accounting for habitat covariates as separately affecting the observation and the state processes. Indeed when compared to the canonical approach to distance sampling, the method we used refined density estimation differences among forests. This is of particular relevance to populations in highly impacted forests as US, where animals can go easily undetected and are unevenly located within the sampled area; more generally, it represents a valuable tool for the study of threatened and/or low density populations, as failure to model covariates of detectability and abundance will likely produce biased density estimates. We also showed that group size influences the observation process and is of particular importance for species or populations with small social units. Lastly, this approach allows spatially-explicit modelling of animal density at the scale of the covariates used in the modelling. Hence, when significant covariates are available across the study area (forest blocks in our case), and even beyond, such as from remote sensing layers (e.g. elevation, slope, distance to disturbances, etc.) inference on density can be extended over such areas (hence even beyond the measurement points), providing a critical tool to predict the status of populations in fragmented or otherwise heterogeneous landscapes.

## Supporting Information

S1 File Best selected model detection functions for RC (Figure A), covariates effect on density estimation shown for BW and SY (Figure B) and spatially explicit modelling of animal density in MG, MT and US (Figure C). (PDF)
