# Supplementary material for: Primates in Human-Modified and Fragmented Landscapes: The Conservation Relevance of Modelling Habitat and Disturbance Factors in Density Estimation
Source: PLoS One. 2016 Feb 4;11(2):e0148289. doi: 10.1371/journal.pone.0148289 (PMC4742242; doi:10.1371/journal.pone.0148289)
Supplement: S1 File — (PDF) [file pone.0148289.s001.pdf]

Figure A. Detection functions from the best AIC models, shown for the 0.25, 0.50 and 0.75 quartiles of the covariates "distance from disturbance" and "climber percentage" for the Udzungwa red colobus (RC).

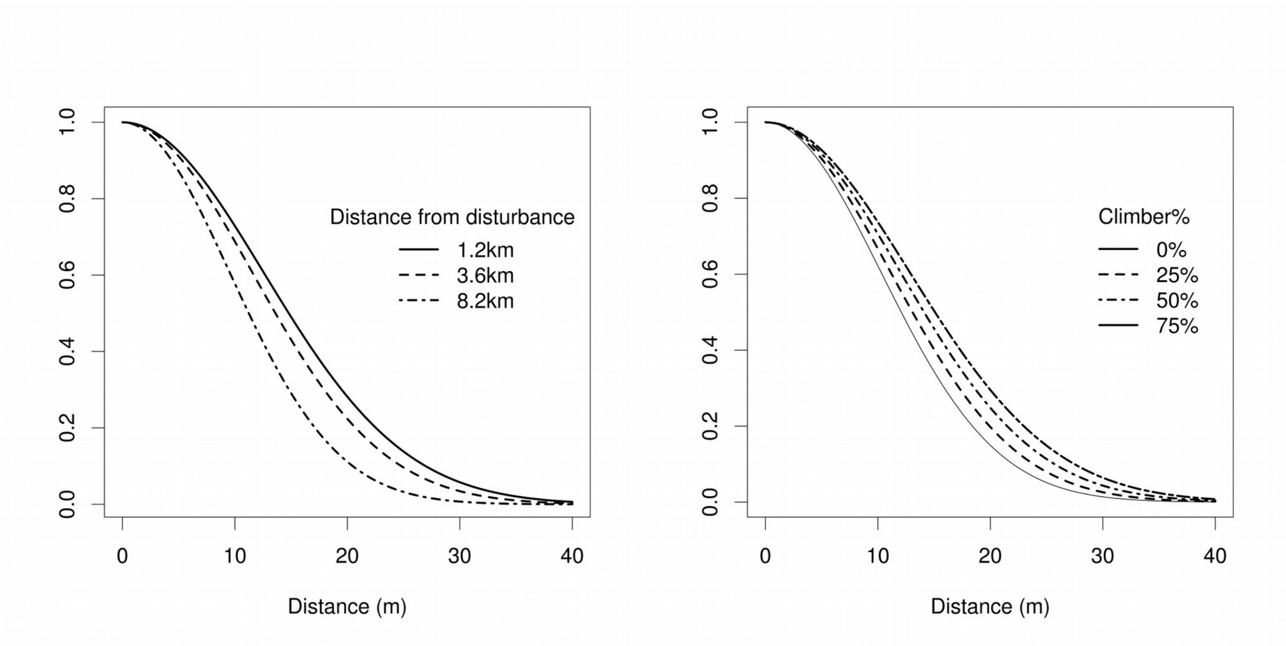

Figure B. Covariates effect on group density estimation, shown for the best model selected for (a) Peters' Angola colobus (BW) and (b) Tanzania Sykes' monkey (SY) .

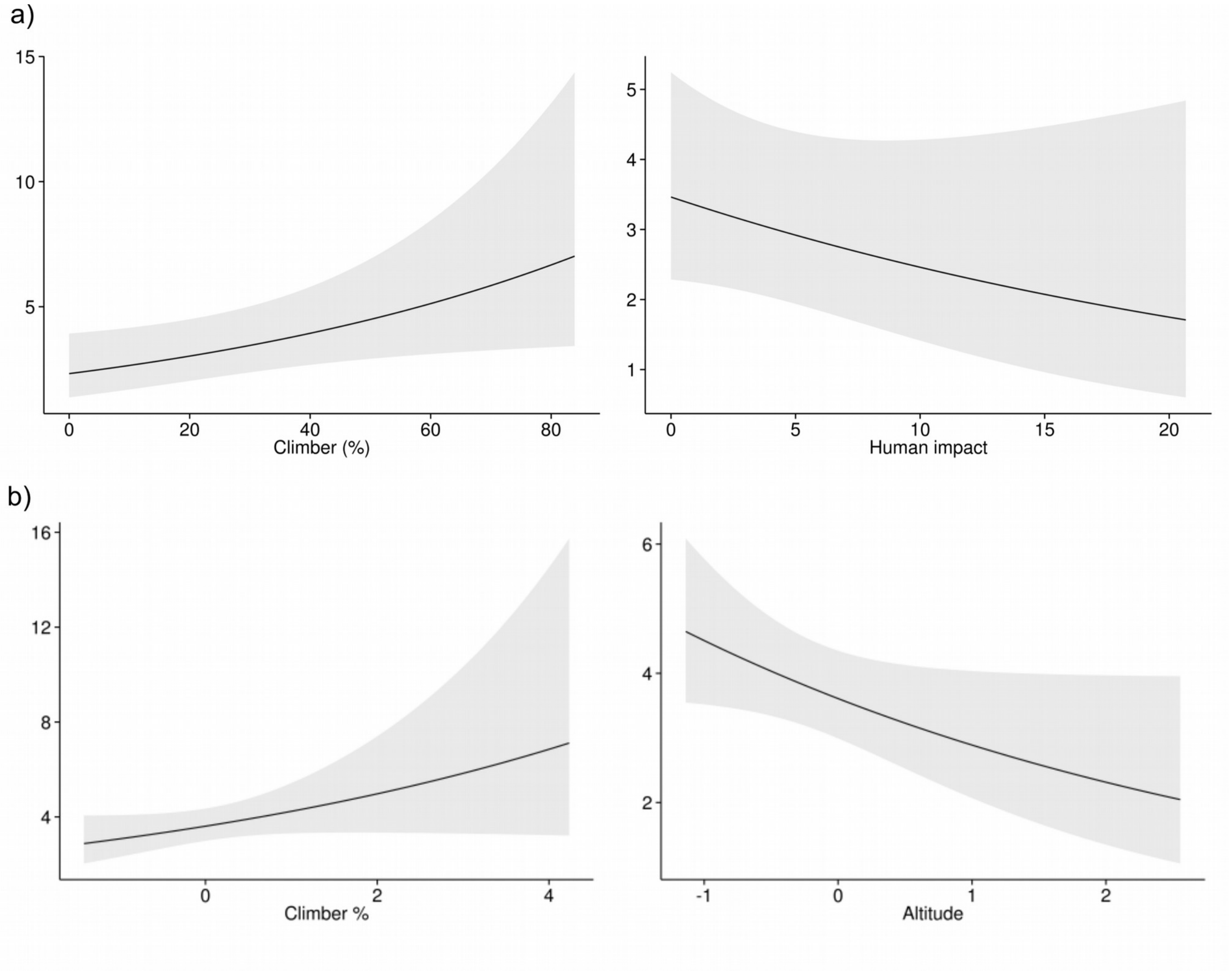

Figure C. Predicted density (groups/km2) for the three primate species (Peters' Angola colobus, Udzungwa red colobus, Tanzania Sykes' monkey) from the best selected models (see Table 3) in the forest of (a) Magombera, (b) Matundu and (c) Uzungwa Scarp.

a)

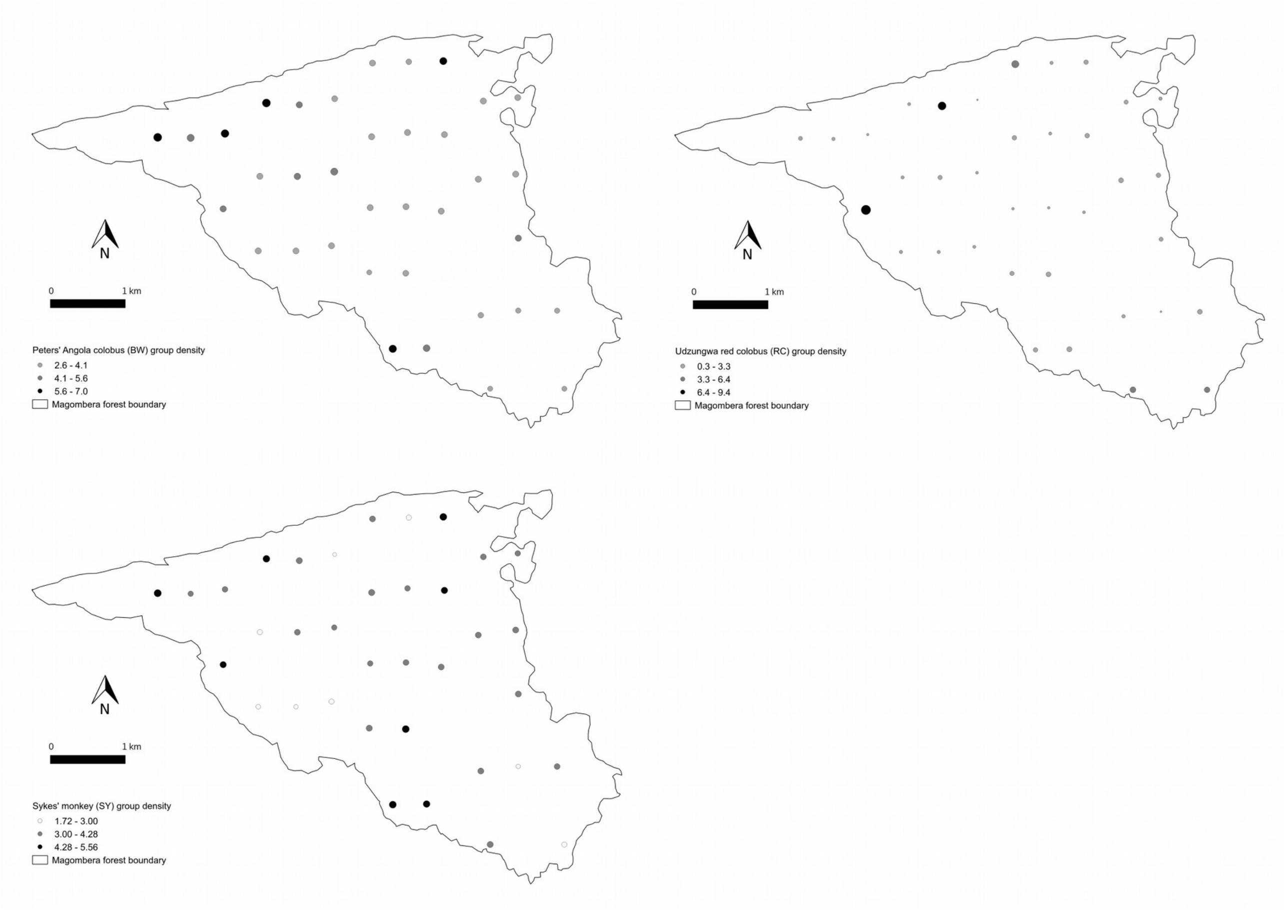

b)

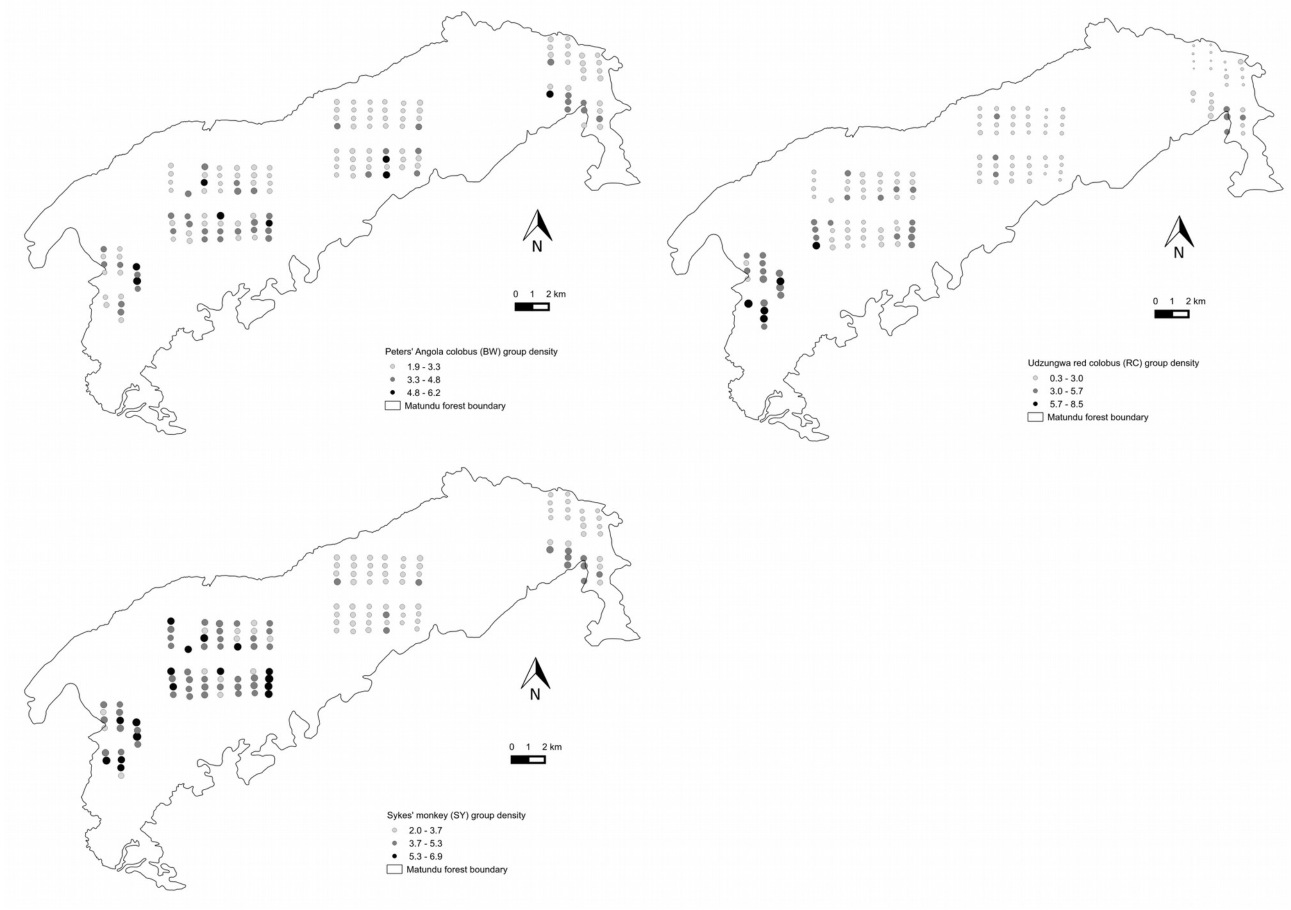
N
0 1 2 km
Peters' Angola colobus (BW) group density
1.9 - 3.3
3.3 - 4.8
4.8 - 6.2
Matundu forest boundary
N
0 1 2 km
Udzungwa red colobus (RC) group density
0.3 - 3.0
3.0 - 5.7
5.7 - 8.5
Matundu forest boundary
N
0 1 2 km
Sykes' monkey (SY) group density
2.0 - 3.7
3.7 - 5.3
5.3 - 6.9
Matundu forest boundary

c)

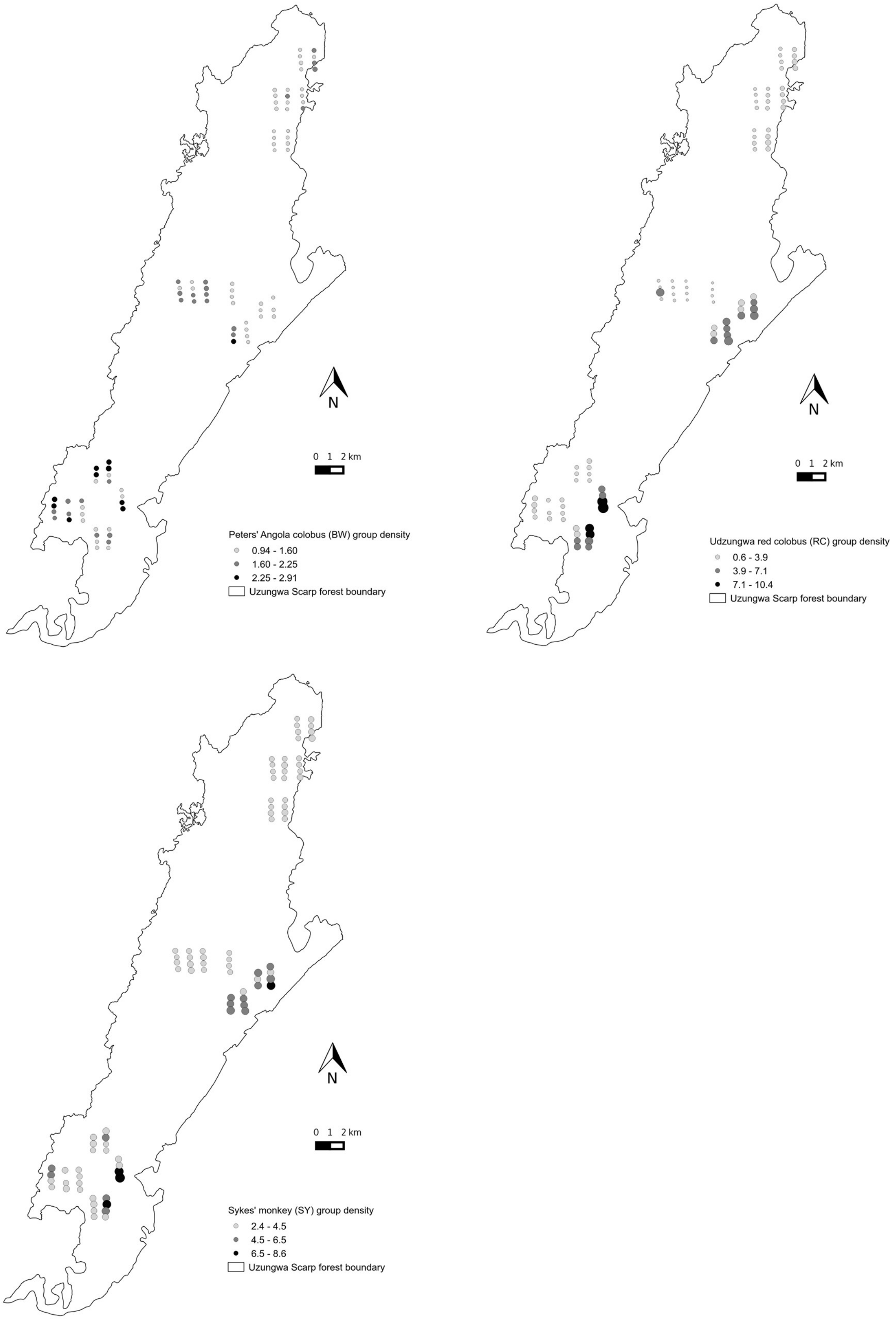
N
0 1 2 km
Peters' Angola colobus (BW) group density
0.94 - 1.60
1.60 - 2.25
2.25 - 2.91
Uzungwa Scarp forest boundary
N
0 1 2 km
Udzungwa red colobus (RC) group density
0.6 - 3.9
3.9 - 7.1
7.1 - 10.4
Uzungwa Scarp forest boundary
N
0 1 2 km
Sykes' monkey (SY) group density
2.4 - 4.5
4.5 - 6.5
6.5 - 8.6
Uzungwa Scarp forest boundary
